# Molecular, Physiological and Biochemical Responses of *Theobroma cacao* L. Genotypes to Soil Water Deficit

**DOI:** 10.1371/journal.pone.0115746

**Published:** 2014-12-26

**Authors:** Ivanildes C. dos Santos, Alex-Alan Furtado de Almeida, Dário Anhert, Alessandro S. da Conceição, Carlos P. Pirovani, José L. Pires, Raúl René Valle, Virupax C. Baligar

**Affiliations:** 1 Departamento de Ciências Biológicas, Universidade Estadual de Santa Cruz, Campus Soane Nazaré de Andrade, Rod. Jorge Amado, km 16, 45662-900, Ilhéus, Bahia, Brazil; 2 Centro de Pesquisas do Cacau, Comissão Executiva do Plano da Lavoura Cacaueira (CEPEC/CEPLAC). Rod. Jorge Amado, km 22, 45650-000, Ilhéus, Bahia, Brazil; 3 United States Department of Agriculture, Agricultural Research Service, Beltsville, Maryland, 20705-2350, United States of America; University of Antwerp, Belgium

## Abstract

Six months-old seminal plants of 36 cacao genotypes grown under greenhouse conditions were subjected to two soil water regimes (control and drought) to assess, the effects of water deficit on growth, chemical composition and oxidative stress. In the control, soil moisture was maintained near field capacity with leaf water potentials (ΨWL) ranging from −0.1 to −0.5 MPa. In the drought treatment, the soil moisture was reduced gradually by withholding additional water until ΨWL reached values of between −2.0 to −2.5 MPa. The tolerant genotypes PS-1319, MO-20 and MA-15 recorded significant increases in guaiacol peroxidase activity reflecting a more efficient antioxidant metabolism. In relation to drought tolerance, the most important variables in the distinguishing contrasting groups were: total leaf area per plant; leaf, stem and total dry biomass; relative growth rate; plant shoot biomass and leaf content of N, Ca, and Mg. From the results of these analyses, six genotypes were selected with contrasting characteristics for tolerance to soil water deficit [CC-40, C. SUL-4 and SIC-2 (non-tolerant) and MA-15, MO-20, and PA-13 (tolerant)] for further assessment of the expression of genes NCED5, PP2C, psbA and psbO to water deficit. Increased expression of NCED5, PP2C, psbA and psbO genes were found for non-tolerant genotypes, while in the majority of tolerant genotypes there was repression of these genes, with the exception of PA-13 that showed an increased expression of psbA. Mutivariate analysis showed that growth variables, leaf and total dry biomass, relative growth rate as well as Mg content of the leaves were the most important factor in the classification of the genotypes as tolerant, moderately tolerant and sensitive to water deficit. Therefore these variables are reliable plant traits in the selection of plants tolerant to drought.

## Introduction

Cacao (*Theobroma cacao* L.) is a perennial crop of great economic importance grown in tropical regions of the world to produce cocoa beans used mainly for the manufacture of chocolate [1]. The species originated in the Amazon region [2] but was initially domesticated in Central America by the Mayas, approximately 3,500 years ago [3]. There are three main cacao groups, Criollo, Forastero and Trinitario, distinguished by their botanical features and geographic origins [4].

Although cacao is typically grown in areas of high annual rainfall [5], the growing regions are prone to irregular rainfall and a range of drought conditions. Furthermore, in some growing areas low water storage capacity of the soil is one of the main causes of irregularity in annual production. Therefore, cacao production is affected by soil water deficiency in some parts of the world [6], [7]. Like other plants, cacao plants have adapted several survival mechanisms under drought conditions, which can be exploited to identify drought tolerant genotypes that maintain good productivity under conditions of low soil water availability [8]. When subjected to water stress, plants exhibit: (i) inhibition of growth and development, (ii) changes in the roots/shoot ratio and increases in biomass allocation to roots rather than shoots [9], (iii) increases in root length which facilitate the exploration of larger soil volumes, and consequently increases water and nutrients absorption [10], [8], (iv) production of reactive oxygen species (ROS) [11], [12], (v) changes in the activity of enzymes involved in the antioxidant metabolism [13], (vi) differential gene expression [14] and (vii) changes in the absorption kinetics of mineral nutrients [15].

Mineral nutrients are involved in several biochemical mechanisms, including signal transduction, enzyme activation, plant growth and the photosynthetic process [16]. A deficit of water in the soil impairs the availability of nutrients and their subsequent uptake by roots [15] and may alter biomass allocation to the root system as a result of metabolic changes in the shoots. It also interferes with carbohydrates transport to the roots [17] and distribution of nutrients to the shoots [18]. On the other hand, changes in the macronutrient and micronutrient concentrations in plant may confer better survival conditions of plants under stress [15].

The Ca^2+^ ion, a secondary messenger in signal transduction pathways, generally increased concentrations in response to stress signals [19], [20], which may lead to an increase in abscisic acid (ABA) concentrations [20]. K^+^ and anion efflux mediate stomatal closure [21], [20], [22] and serve as osmoregulators, maintaining plant turgidity under drought conditions [10]. Theplants supplied with adequate P and subjected to water stress show an increase in photosynthetic efficiency and in the activity of oxidative stress enzymes, resulting in an increase in biomass [23]. Additionally, under conditions of low soil water availability there may be a shortage of Mg^2+^ and alteration in the biomass allocation from roots to shoots [24].

In general, ROS production intensifies when plants are subjected to biotic and abiotic stresses, resulting in oxidative stress [11], [12]. Antioxidative metabolism enzymes use Zn, Cu and Mn as cofactors [1], [18]. Changes in the activity of these enzymes to remove ROS increase the plant's drought tolerance [25], [13], [26]. In addition, ROS play a fundamental role in the regulation of gene expression [27], [28], perception and signal transduction [29].

Perception and signal transduction by plants under water stress conditions are driven by two distinct pathways, the ABA dependent and independent routes. During abiotic stress, ABA may be synthesized via the carotenoids biosynthetic pathway, in which the cleavage of cis-xanthophylls is catalyzed by a family of 9-cis-epoxicarotenoide dioxygenases (NCED) [30], [31], and acts as a messenger in endogenous stress responses [32], [33]. In addition, some genes are negative regulators of ABA-dependent pathways, such as the family *PP2C*, encoding phosphatases, which in turn inhibit kinases and thus gene expression, and promote activation of anion (SLAC1) and cation channels [34].

In addition to genes known to be involved with water stress tolerance, over expression and/or repression of those involved in biosynthetic proteins routes, especially the pathway associated with carbon assimilation, are of great importance since they are related to the yield production of cultivated species [35]. The D1 protein, encoded by *psbA*, a component of PSII involved in photosynthetic electron transport, can be easily degraded and is continuously synthesized under stress [35]. On the other hand, the *psbO* protein, involved in the stabilization and oxygen evolution in the Mn cluster at PSII, has a fundamental role in photosynthesis [36] and performs a protective function for photosynthetic apparatus during abiotic stresses [37]. However, the high stability of PSII during drought observed in *Festuca arundinacea*, a highly drought tolerant species, is not associated with the accumulation of *psbO*, although its degradation affects the destabilization of the oxygen evolution complex under drought conditions [37].

The objectives of this study were to evaluate growth, chemical composition and oxidative stress of a sample of 36 cacao genotypes of different geographical origins subjected to water stress (drought). Also, to evaluate the expression of genes related to drought tolerance and biosynthesis of *psbO* and *psbA* proteins in genotypes identified in this study as tolerant and non-tolerant to water stress, aiming to elucidate possible mechanisms of drought tolerance and offer support for selection of genotypes to be grown in soils with low water storage capacity and/or in regions with irregular rainfall.

## Materials and Methods

### Plant material and growth conditions

A sample of 36 cacao genotypes, belonging to genetic groups Forastero, Criollo and Trinitario was selected for this study (Table 1). As no information is available regarding the level of drought resistance of these genotypes, we selected original clonal accessions collected from different geographical regions used as progenitors in breeding programs and hybrids to compose the sample. Seminal seedlings were prepared from open pollinated seeds collected from clonal accessions at the Cacao Germplasm Bank of the Cacao Research Center (CEPEC), the research facility of the Executive Commission of the Cacao Farming Plan (CEPLAC), Ilhéus, Bahia. Five fruits were collected from each of the 36 genotypes, the seeds of each genotype were mixed and a randomly composed sample of 40 seeds were planted in 16 L pots containing soil as the substrate. Chemical and physical analyses of the soil were performed and fertilized according to the crop requirements during the seedling production [38]. The experiment was conducted in a greenhouse at CEPEC/CEPLAC, Ilhéus, Bahia, Brazil (14°47'S, 39°16'W, 55 m ASL).

**Table 1:** List of 36 cacao genotypes subjected to soil water deficit and their geographical origin, botanical group and gametic compatibility.

Genotype	Origin	Botanical group	Gametic compatibility
AMAZON -15.1 (AMZ-15.1)	Peru	Forastero	Self-incompatible
BE- 08	Brazil	Forastero	Self-compatible
C SUL-3	Brazil	Forastero	Self-incompatible
C SUL-4	Brazil	Forastero	Self-incompatible
CA-1	Brazil	Forastero	-
CA-3	Brazil	Forastero	-
CAB-139	Brazil	Forastero	-
CAB-274	Brazil	Forastero	-
CATONGO (CAT)	Brazil	Forastero	Self-compatible
CC-40	Costa Rica	Hybrid	Self-compatible
EET-103	Ecuador	Hybrid	-
EET-53	Ecuador	Hybrid	Self-compatible
EQX-107	Ecuador	Hybrid	-
GU-114	French Guiana	Forastero	-
ICS-9	Trinidad	Trinitario	Self-compatible
ICS-98	Trinidad	Trinitario	Self-incompatible
IMC-27	Peru	Forastero	-
IMC-76	Peru	Forastero	Self-incompatible
MA-14	Brazil	Forastero	Self-incompatible
MA-15	Brazil	Forastero	Self-incompatible
MO-20	Peru	Forastero	-
MOCORONGO 2 (MOC-2)	Brazil	Forastero	-
OC-77	Venezuela	Criollo	Self-compatible
PA-13	Peru	Forastero	Self-incompatible
PA-150	Peru	Forastero	Self-incompatible
PS-1319	Brazil	Complex hybrid	Self-compatible
RB-39	Brazil	Forastero	Self-incompatible
RB-48	Brazil	Forastero	Self-incompatible
RIM-6	Mexico	Criollo	Self-incompatible
SCA-6	Peru	Forastero	Self-incompatible
SIAL-169	Brazil	Forastero	Self-compatible
SIC-17	Brazil	Forastero	Self-compatible
SIC-2	Bahia	Forastero	Self-compatible
SPA-5	Colombia	Forastero	Self-compatible
TSA-792	Trinidad	Hybrid	Self-incompatible
TSH-1188	Trinidad	Hybrid	Self-incompatible

**Figure 1:**
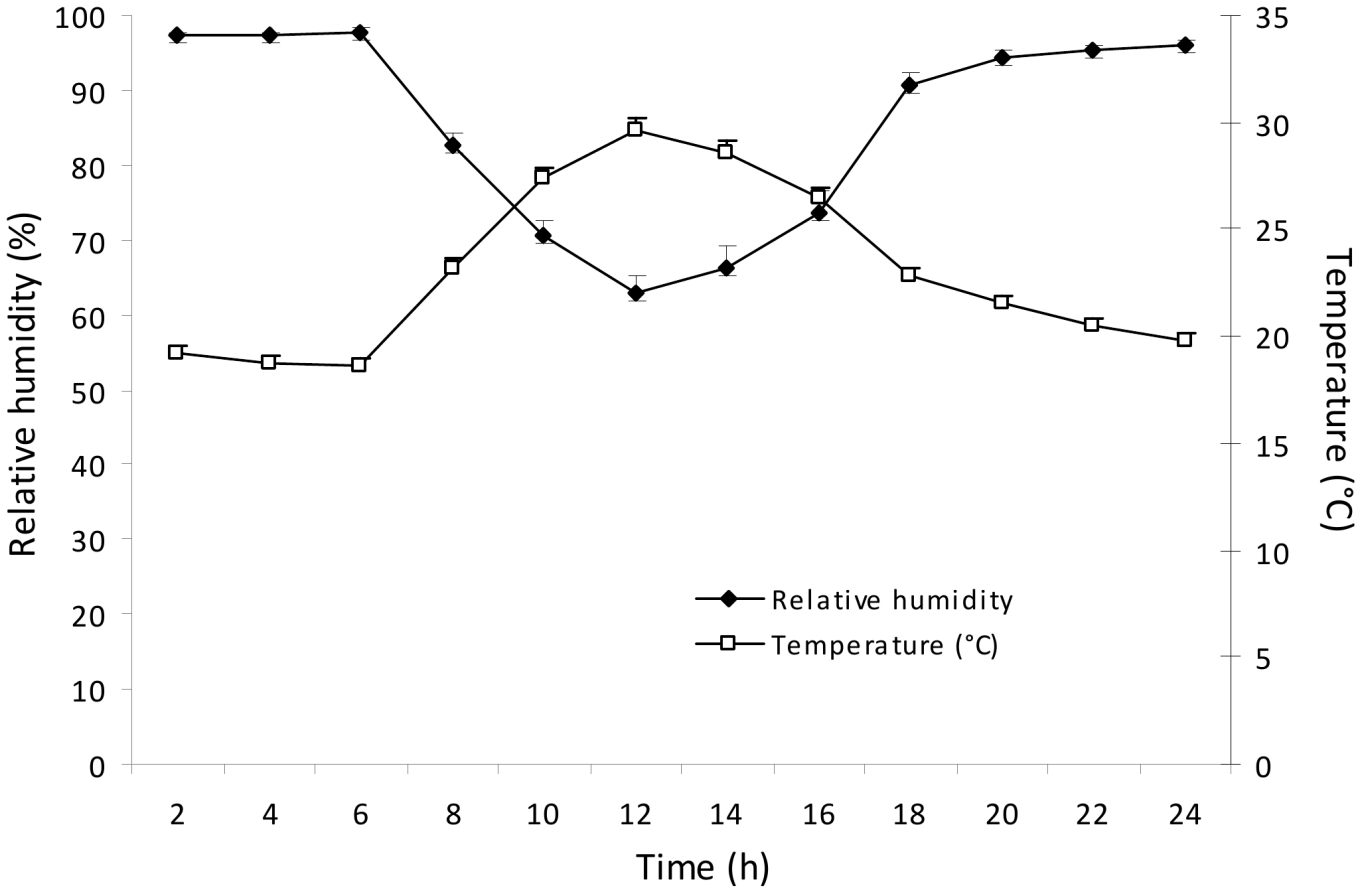
Average daytime temperature and relative humidity of the air during the trial period. Average values of 60 days ± standard error.

During the time of the experiment, temperature and relative humidity were recorded (Fig. 1) using a thermo-hygrograph (Kipp & Zonen, model 836); and photosynthetically active radiation (PAR) was measured using a quantum meter (Model-QMSS SUN-1350 Apogee, City, USA). The maximum values of PAR inside the greenhouse ranged from 800 to 1200 µmol photons m^−2^ s^−1^. Six months-old plants were divided into two groups and one group was subjected to drought by gradually reducing the soil water content by reducing water addition until the dawn leaf water potential (Ψ_WL_) reached −2.0 to −2.5 MPa, these leaf water potentials were reached approximately 40–60 days after the beginning of the drought cycle. The second group of plants were used as controls and irrigated daily to maintain soil moisture near field capacity and Ψ_WL_ between −0.1 to −0.5 MPa. Measurements of Ψ_WL_ were done at the second or third mature leaf from the apex of the orthotropic axis between 2:00 and 4:00 am, using a pressure chamber (Model 1000, PMS Instrument Company, Albany, OR, USA) [39].

### Growth parameters

Plant samples were collected at the beginning of the drought cycle (six months-old plants), when the Ψ_WL_ of all genotypes was between −0.1 to −0.5 MPa and the soil moisture was near field capacity, and at 40 to 60 days after the beginning of the drought cycle when the Ψ_WL_ of the different genotypes reached −2.0 to −2.5 MPa. Just before harvest, measurements were made for total leaf area per plant (TLAP), stem diameter (SD), plant height (PH), and leaf number per plant (LNP). The features SD and PH were measured using a digital caliper and ruler, respectively. At harvest the plants were divided into roots, stem and leaves.

Leaf area was measured by Li-Cor model Li-3100 leaf area meter (Li-Cor, inc. Lincoln, Nebraska, USA). Root area (ARS) was estimated after limiarization in the Gimp 2 software and subsequent analysis with the Sigma Scan Pro 5 program and root volume was estimated after immersion of roots in a known water volume and observing its displacement. Different plant parts were placed in paper bags and dried at 75 °C in a forced air circulation over to obtain total dry mass of the plant and its parts. From the dry biomass data of the different plant parts (root-RDB, stem-SDB, leaf-LDB) and total leaf area per plant (TLAP) several indices were determined for all genotypes: (i) accumulation of total biomass (TDB), (ii) relative growth rate [RGR =  (ln TDB_2_ - ln TDB_1_)/(T_2_-T_1_)], (iii) net assimilation rate {NAR =  [(TDB_2_ - TDB_1_)/(TLAP_2_ - TLAP_1_)] × [(ln TLAP_2_ - ln TLAP_1_)/(T_2_ - T_1_)]}, (iv) leaf number per plant (LNP), leaf area ratio (TLAP/TDB), (v) individual leaf area (ILA =  TLAP/LNP), (vi) specific leaf biomass (SLB =  LDB/TLAP) (vii) shoot dry biomass (SB =  LDB + SDB) and (viii) root/shoot ratio (R/S) [40], [41], [42].

### Macro and micro mineral nutrients

The leaf content of mineral macro and micronutrients was determined in all 36 genotypes studied. Approximately 200 mg of ground dry biomass was used for nitropercloric digestion (3∶1). After digestion, Ca, Mg, Fe, Zn, Cu and Mn values were determined by atomic absorption spectrophotometery, P by colorimetry and K by flame emission photometry [43]. Nitrogen was determined by the Kjeldahl method after sulphosalicylic digestion [44]. Leaf mineral content was expressed as g plant^−1^ for each genotype and treatment.

### Oxidative stress

The activities of guaiacol peroxidases (GPX-EC1.11.1.7) and polyphenol oxidases (PPOs, EC1.10.3.1) were determined in leaf samples collected from the second and third mature leaf from the apex of the orthotropic axis of all cacao genotypes. The samples were immersed in liquid nitrogen, stored in a freezer at −80°C and subsequently lyophilized. Extraction of enzymes and determination of their activities were performed following methodology described by Pirovani *et al.* [45]. Conversion of absorbance data (470 nm min^−1^ g^−1^ DW) to guaiacol consumption in mmol g^−1^ DW h^−1^ was performed using the equation y = 0.1324+0.8382× (r^2^ = 0.99), while conversion for the PPOs data from absorbance (444 nm min^−1^ g^−1^ DW) to epicatechin consumption in mg g^−1^ DW min^−1^ was performed through the equation y = 50.657×+0.091 (r^2^ = 0.99). The readings were performed in a microplate reader VERSAmax (Tunable Molecular Devices, Sunnyvale, CA, USA).

### Gene expression

RNA was extracted from the second or third mature leaf from the apex of the orthotropic axis of six cacao genotypes [CC-40, C. SUL-4 and SIC-2 (non-tolerant) and MA-15, MO-20, and PA-13 (tolerant)], identified during the data analysis. The leaf samples were immersed in liquid nitrogen, stored at −80°C and subsequently lyophilized for gene expression analyses. For this study we used four genes: two candidate genes related to drought tolerance, involved in the ABA dependent pathway, *NCED5* (9-cis-epoxycarotenoid dioxygenase 5) and *PP2C* (protein phosphatase-2C) and two genes related to proteins biosynthesis of PS II (*psbA* and *psbO*) (Table 2).

**Table 2:** Gene specific pairs of primers used in qPCR analysis.

Gene	Accession no.	*Primer*
*NCED5*	TC09:23395416..23395838 *	*Forward*; 5′- CAGACATTTTCAGGACTTCTTCA -3′
		*Reverse*; 5′-TGGAGCGTTCCATAAACACTTG -3′
*PP2C*	CL5350Contig1 **	*Forward*; 5′-TGCTGAAGATCAAAATTGGTTAGG-3′
		*Reverse*; 5′-GGAAAAGATAAGCATGAAGTGG-3′
*PsbO*	CL326Contig1**	*Forward*; 5′-GCAAACGCTGAAGGAGTT-3′
		*Reverse*; 5′-GGCTTGAAGGCAAATGAGTC-3′
*PsbA*	NC_014676.2 ***	*Forward*; 5′-GGTTTGCACTTTTACCCGA-3′
		*Reverse*; 5′- CTCATAAGGACCGCCATT -3′
*β-Tubulina*	GU570572.1***	*Forward*; 5′-TGCAACCATGAGTGGTGTCA- 3′
		*Reverse*; 5′-CAGACGAGGGAAAGGAATGA- 3′

* http://cocoagendb.cirad.fr/;

** http://esttik.cirad.fr/index.html;

*** http://www.ncbi.nlm.nih.gov/.

Approximately 0.02 g of each leaf sample was macerated in liquid nitrogen for RNA extraction with the RNAqueous kit (Ambion) following the manufacturer's recommendations. Samples of RNA were used for first-strand cDNA synthesis with RevertAid H Minus M-MuLV Reverse Transcriptase (Fermentas), according to the manufacturer's instructions using oligo d(T)_18_ primers. The reactions were incubated at 65°C for 5 min, 37°C for 5 min, 42°C for 60 min and 70°C for 10 min. The primers were designed after analysis of conserved sequences in *T. cacao* (Table 2). The q-PCR was performed in a RT-PCR thermocycler (Applied Biosystems, 7500 model) using the nonspecific detection sequence (fluorophore) SYBR Green I. The mix for the reaction was composed of cDNA as template, 0.5 µM of each primer and 12.5 µL of Maxima SYBR Green/ROX qPCR Master Mix 2x. Quantification of relative expression of genes were calculated as a percentage of the control treatment using the 2^–ΔΔCt^ method [46] and the β-tubulin as endogenous control in order to detect changes in transcript number (Table 2).

### Multivariate analysis

Principal component and cluster analyses were performed using growth variables, chemical composition and oxidative stress values, obtained by the difference (Δ) between control plants (−0.1 to −0.5 MPa) and plants subjected to soil water deficit (−2.0 to −2.5 MPa). Initially, the 28 variables (TLAP, LNP, ILA, RDB, SDB, LDB, SB, TDB, SLB, R/S, HP, ARS, RV, SD, LAR, RGR, NAR, GPX, PPO, leaf contents of N, P, K, Ca, Mg, Fe, Zn, Cu and Mn) were standardized as we measured them in different units (g, cm as well as ratios between them). The standardization was performed by the equation: Zij  = (Xij -Xj)/Sj, where Xij is the value of the i-th observation of the variable Xj; and Xj and Sj is the mean and standard deviation of the variable Xj, respectively. The 28 standardized variables were submitted to cluster analysis and factor analysis, using Statistica version 7 (Statsoft, Inc.Tulsa, OK, USA.). Nine of those variables made the greatest contribution to the formation of the first factor of the factorial analysis. These variables were submitted to colinearity analysis, based on tolerance and on the variance inflation factor (VIF), considering greater than 0.1 and less 10 [47], respectively, as the threshold for variable inclusion in the cluster and principal component analysis, using SPSS (SPSS, Inc., Chicago, IL). From the colinearity analysis it was found that eight variables were not collinear (TLAP, RDB, SDB, LDB, TDB, RGR, and leaf contents of Ca and Mg). These variables were used for cluster and principal components analyses. Cluster analysis was performed based on Euclidean distance and the dendograms constructed using the hierarchical agglomerative method [48].

### Statistical analysis

We used a completely randomized design with 144 treatments [36 genotypes, two water regimes (control - Ψ_WL_ between −0.1 to −0.5 MPa and drought - Ψ_WL_ between −2.0 to −2.5 MPa) and two sampling times of plant material - baseline and 60 days of stress] and six replications (plants) for collecting RGR and NAR variables; with 72 treatments (36 genotypes and two water regimes) and six to eight replications to assess growth, oxidative stress and chemical composition; and with 12 treatments [six genotypes and two water regimes] and four replications (grounded pooled leaves of two plants) for gene expression assessment. Results were subjected to comparisons of treatment means using the Student t-test (P<0.05 and 0.01). Based on the results of the Student t-test we grouped the genotypes into three types: (i) tolerant genotypes, those that had from 0 to 10 significant variables; (ii) moderately tolerant genotypes, those that had from 11 to 15 significant variables; and (iii) sensitive genotypes, those that had above 16 significant variables.

## Results

### Accumulation and partitioning of dry biomass

Soil water deficit significantly (P<0.05) influenced biomass production, reducing dry weight in all plant parts for most of the evaluated cacao genotypes, except EET-53, ICS-9, MA-15, OC-77, PA-150, PS-1319 and SPA-5 (Table 3). Significant reductions (P<0.05) in root (RDB), stem (SDB), leaf (LDB), shoot (SB) and total (TDB) dry biomass were found in 42, 50, 50, 58 and 64% of the genotypes, respectively, in relation to their controls, for each of these variables. Decreases in LDB, SB, SDB and RDB were observed mainly in drought sensitive genotypes (Table 3).

**Table 3:** Growth and biomass characteristics of cacao genotypes subjected to two water regimes.

Genotype	Treatment	TLAP ×10^3^	LNP	ILA ×10^−2^	RDB	SDB	LDB	SB	TDB	SLB	R/S	HP	LAR	NAR	RGR	ARS	RV	SD
AMZ 15.1	Control	95±6	37±2	2.6±0.0	30±4	72±8*	48±3	120±8**	149±5**	51±2	0.3±0.1	135±6*	0.6±0.0	0.0±0.0*	0.0±0.0*	480±28**	102±15	21±1
	Drought	79±4	32±4	2.5±0.2	25±3	47±2	38±4	84±3	110±5	48±5	0.3±0.0	113±3	0.7±0.1	0.0±0.0	0.0±0.0	192±9	83±13	19±1
BE- 08	Control	86±4**	43±3*	2.0±0.1	27±2**	41±3	43±2**	84±4*	111±5*	50±1	0.3±0.0	132±2	0.8±0.0	0.0±0.0	0.0±0.0	710±48**	127±17 *	19±1
	Drought	64±6	35±2	1.8±0.1	19±2	40±5	32±3	72±7	91±7	50±1	0.3±0.0	122±5	0.7±0.0	0.0±0.0	0.0±0.0	299±35	74±10	19±0
CSUL- 3	Control	95±5*	45±3	2.2±0.1	26±3*	53±5**	51±3*	104±6**	130±7**	54±1	0.3±0.0	115±1	0.7±0.0	0.0±0.0**	0.0±0.0**	393±36**	109±10 **	20±1
	Drought	74±7	35±3	2.1±0.2	17±1	35±2	40±4	75±5	93±6	54±1	0.2±0.0	108±6	0.8±0.0	0.0±0.0	0.0±0.0	143±22	50±4	17±1
CSUL -4	Control	117±6**	52±4**	2.3±0.1*	23±3*	44±4**	55±3**	99±7**	122±8**	47±1	0.2±0.0	142±5**	1.0±0.1	0.0±0.0**	0.0±0.0**	387±34**	94±9 **	19±0 **
	Drought	66±6	33±3	2.0±0.1	15±2	28±3	33±3	60±5	75±7	49±1	0.2±0.0	116±5	0.9±0.0	0.0±0.0	0.0±0.0	190±50	51±7	16±1
CA-1	Control	98±4*	52±2*	1.9±0.1	27±2	46±2**	42±2	88±3**	115±5**	43±3	0.3±0.0	133±4	0.9±0.1	0.0±0.0**	0.0±0.0**	524±28**	117±8 **	20±1 **
	Drought	85±3	45±2	1.9±0.1	21±2	34±2	33±4	67±4	88±4	40±5	0.3±0.1	133±3	1.0±0.0	0.0±0.0	0.0±0.0	376±20	77±2	17±0
CA-3	Control	84±5	56±4*	1.5±0.1*	24±3	48±3**	41±3	89±4**	114±4	49±1	0.3±0.0	140±5*	0.7±0.0	0.0±0.0	0.0±0.0	504±6**	122±8 **	21±0 **
	Drought	72±6	40±4	1.8±0.1	24±6	32±3	34±3	66±6	90±11	47±1	0.3±0.1	124±5	0.8±0.1	0.0±0.0	0.0±0.0	280±34	65±5	17±1
CAB-139	Control	116±5**	49±4**	2.4±0.2	27±4*	56±3**	59±2**	115±4**	142±7**	51±1	0.2±0.0	136±9	0.8±0.0	0.0±0.0*	0.0±0.0**	233±10	96±13 *	21±1 *
	Drought	80±7	33±1	2.4±0.1	16±0	37±4	39±3	76±7	93±7	49±2	0.2±0.0	118±8	0.9±0.1	0.0±0.0	0.0±0.0	200±22	61±4	19±0
CAB-274	Control	106±5*	49±3	2.2±0.0*	21±3	48±5	50±2**	99±6*	120±8*	48±1	0.2±0.0	147±7	0.9±0.0	0.0±0.0*	0.0±0.0*	352±40*	99±9 **	20±0 **
	Drought	84±5	41±2	2.0±0.1	16±1	39±2	38±2	78±3	93±4	46±1	0.2±0.0	141±5	0.9±0.1	0.0±0.0	0.0±0.0	208±18	55±4	17±0
CAT	Control	85±4**	41±3**	2.1±0.0	22±2	40±2*	42±3**	82±2**	103±3**	49±1	0.3±0.0	121±7	0.8±0.0	0.0±0.0	0.0±0.0	378±24**	88±6	18±0
	Drought	58±5	27±1	2.2±0.2	20±2	34±2	30±2	64±3	84±2	52±2	0.3±0.0	112±4	0.7±0.1	0.0±0.0	0.0±0.0	240±17	74±5	18±0
CC-40	Control	112±7**	59±5**	1.9±0.1*	26±3*	51±3**	53±3**	104±4**	130±5**	47±2	0.2±0.0	134±9	0.9±0.1	0.0±0.0**	0.0±0.0**	411±35**	101±9 **	21±1 **
	Drought	73±3	42±3	1.7±0.1	19±1	34±1	33±1	67±2	85±2	45±1	0.3±0.0	117±7	0.9±0.1	0.0±0.0	0.0±0.0	269±15	63±3	18±0
EET-103	Control	91±11	40±5	2.3±0.1	19±2	56±6	43±5*	99±11	118±11	47±2	0.2±0.0	139±9	0.8±0.1	0.0±0.0	0.0±0.0	352±27*	79±10	20±1
	Drought	70±6	32±3	2.2±0.3	18±2	42±6	31±2	73±7	92±8	45±3	0.3±0.0	123±5	0.8±0.0	0.0±0.0	0.0±0.0	243±25	65±4	19±1
EET-53	Control	80±3	41±3	2.0±0.1	23±2	39±4	38±1	77±5	100±5	48±2	0.3±0.0	123±5	0.8±0.1	0.0±0.0	0.0±0.0	389±34	103±9 *	20±1
	Drought	69±7	37±2	1.9±0.2	19±2	34±2	33±2	67±3	86±4	49±2	0.3±0.0	122±4	0.8±0.1	0.0±0.0	0.0±0.0	243±26	69±7	19±1
EQX-107	Control	107±11	50±8	2.2±0.2	28±1**	59±3*	52±5	111±8*	139±8*	49±1	0.3±0.0	139±3	0.8±0.0	0.0±0.0**	0.0±0.0*	655±28**	117±7 **	23±1 **
	Drought	86±9	39±5	2.3±0.2	18±2	42±4	42±4	83±8	101±9	49±1	0.2±0.0	134±8	0.9±0.1	0.0±0.0	0.0±0.0	221±43	63±8	17±1
GU-114	Control	100±4**	53±1**	1.9±0.1	32±3*	58±1**	48±6	106±6**	138±7**	49±6	0.3±0.0	147±8	0.7±0.0	0.0±0.0*	0.0±0.0*	349±27*	137±18 **	21±0 **
	Drought	78±3	39±2	2.0±0.1	20±2	38±3	40±1	78±4	98±5	52±2	0.3±0.0	129±6	0.8±0.1	0.0±0.0	0.0±0.0	248±28	68±6	18±1
ICS-9	Control	88±11*	50±5*	1.7±0.1	18±2	39±4	35±2	74±5	92±6	43±6	0.2±0.0	124±6	1.0±0.1	0.0±0.0	0.0±0.0	289±17**	73±9	18±0
	Drought	56±5	34±2	1.6±0.2	16±2	30±3	31±4	61±6	76±7	51±9	0.3±0.0	117±6	0.8±0.1	0.0±0.0	0.0±0.0	219±9	51±5	16±1
ICS-98	Control	83±7	53±6*	1.6±0.1	24±2*	53±7*	44±3	97±6**	121±7**	54±1	0.3±0.0	128±5	0.7±0.1	0.0±0.0	0.0±0.0	372±46**	93±9 *	19±0
	Drought	61±10	35±2	1.7±0.2	19±1	37±3	33±4	69±6	88±6	55±2	0.3±0.0	123±3	0.7±0.1	0.0±0.0	0.0±0.0	201±19	63±3	18±0
IMC-27	Control	104±5	49±2	2.1±0.1	25±4	42±4	51±3	93±7*	118±11*	49±1	0.3±0.0	128±4	0.9±0.1	0.0±0.0	0.0±0.0*	453±10**	101±12 *	19±1
	Drought	86±9	39±6	2.3±0.2	16±1	33±1	41±4	74±3	90±3	48±1	0.2±0.0	127±4	0.9±0.1	0.0±0.0	0.0±0.0	249±15	65±5	17±1
IMC-76	Control	122±9**	52±2**	2.4±0.1	30±3*	57±4**	55±3**	112±5**	142±6**	45±3	0.3±0.0	140±10	0.9±0.1	0.0±0.0**	0.0±0.0**	680±85**	134±5 **	21±0 **
	Drought	78±6	31±2	2.6±0.1	20±2	37±2	38±2	75±2	95±3	49±5	0.3±0.0	134±8	0.8±0.1	0.0±0.0	0.0±0.0	217±23	69±8	18±1
MA-14	Control	100±9	46±4	2.2±0.2	23±2	46±5*	49±5	95±9	118±#*	49±2	0.2±0.0	129±7	0.8±0.1	0.0±0.0	0.0±0.0	380±14**	93±9 *	20±1
	Drought	82±3	43±3	1.9±0.1	18±2	35±1	39±2	74±2	92±4	48±1	0.2±0.0	128±6	0.9±0.0	0.0±0.0	0.0±0.0	238±29	67±6	18±0
MA-15	Control	96±5	48±3	2.0±0.1	25±3	50±4	45±3	95±7	120±9	47±1	0.3±0.0	137±7	0.8±0.0	0.0±0.0	0.0±0.0	493±46	108±12	20±1
	Drought	80±8	37±5	2.2±0.1	24±3	41±3	38±3	79±4	102±5	48±1	0.3±0.0	120±6	0.8±0.1	0.0±0.0	0.0±0.0	294±10	100±21	18±0
MO-20	Control	90±4*	56±2	1.6±0.1	25±4	55±4	45±2*	100±6*	125±8*	50±2	0.2±0.0	149±6	0.7±0.0	0.0±0.0	0.0±0.0	269±14	98±15	20±1
	Drought	74±5	46±5	1.7±0.1	19±2	44±2	38±3	82±4	101±6	51±2	0.2±0.0	133±5	0.7±0.1	0.0±0.0	0.0±0.0	223±18	67±6	18±1
MOC-2	Control	104±5**	54±3**	1.9±0.1	28±2**	53±4**	52±2	106±4**	133±5**	51±1	0.3±0.0	125±6	0.8±0.1	0.0±0.0*	0.0±0.0**	334±20**	135±27 **	21±0 **
	Drought	78±6	36±2	2.1±0.1	20±1	37±2	41±3	78±3	98±3	53±1	0.3±0.0	116±3	0.8±0.1	0.0±0.0	0.0±0.0	188±6	66±7	17±0
OC-77	Control	42±8	32±6	1.3±0.1	18±3	35±5	22±4	56±8	74±11	52±2	0.3±0.0	104±14	0.6±0.1	0.0±0.0*	0.0±0.0	245±19	66±17	18±2
	Drought	40±6	30±4	1.3±0.2	15±4	24±5	19±3	44±8	58±11	49±1	0.4±0.1	100±12	0.7±0.1	0.0±0.0	0.0±0.0	192±14	47±8	16±1
PA-13	Control	96±5**	53±3**	1.8±0.1	29±3	46±5	47±2**	93±6	122±8	50±2	0.3±0.0	142±7*	0.8±0.1	0.0±0.0	0.0±0.0	341±25	116±15	21±1
	Drought	72±2	36±1	2.0±0.1	28±3	41±4	39±1	80±5	108±7	54±1	0.3±0.0	124±4	0.7±0.1	0.0±0.0	0.0±0.0	287±28	103±12	19±0
PA-150	Control	98±5	55±4*	1.8±0.1	26±2	41±4	46±2	87±4	114±5	48±2	0.3±0.0	129±7	0.9±0.1	0.0±0.0	0.0±0.0	583±45**	138±14 **	19±1 *
	Drought	77±9	41±5	1.9±0.1	22±2	37±2	39±4	76±6	98±6	51±2	0.3±0.0	125±5	0.8±0.1	0.0±0.0	0.0±0.0	262±19	78±8	17±1
PS-1319	Control	94±8	60±5**	1.6±0.1	19±3	39±3	41±4	80±7	99±9	45±6	0.2±0.0	129±4	1.0±0.1	0.0±0.0	0.0±0.0	445±45**	86±12	19±1
	Drought	69±8	37±4	1.8±0.1	17±3	31±4	32±4	63±6	81±8	47±1	0.3±0.0	119±5	0.9±0.1	0.0±0.0	0.0±0.0	244±52	67±6	18±1
RB-39	Control	93±5	44±1	2.1±0.1	24±3	49±4*	48±1	97±4*	121±7	52±2	0.2±0.0	131±5	0.8±0.1	0.0±0.0	0.0±0.0	415±33*	104±8 **	19±1 *
	Drought	90±2	41±4	2.3±0.2	20±2	36±3	45±2	82±5	102±6	51±2	0.2±0.0	128±7	0.9±0.1	0.0±0.0	0.0±0.0	234±55	62±5	17±1
RB-48	Control	101±5**	48±3*	2.1±0.2	27±2**	44±3*	58±2**	102±3**	130±5**	58±1	0.3±0.0	113±3	0.8±0.0	0.0±0.0**	0.0±0.0*	646±40**	132±16 **	21±1 **
	Drought	81±4	39±2	2.1±0.0	18±2	34±2	43±3	77±3	95±4	53±2	0.2±0.0	113±5	0.9±0.0	0.0±0.0	0.0±0.0	262±27	58±7	17±0
RIM-6	Control	93±6*	48±2**	1.9±0.0*	21±3	45±2	49±3*	94±5*	115±7*	53±2	0.2±0.0	135±4	0.8±0.0	0.0±0.0	0.0±0.0	385±24	97±11 *	20±1 *
	Drought	74±5	33±3	2.3±0.1	18±1	39±3	39±2	78±4	96±5	52±1	0.2±0.0	129±5	0.8±0.0	0.0±0.0	0.0±0.0	373±21	63±5	17±1
SCA-6	Control	112±7*	59±5*	1.9±0.1	23±4	57±5	55±2**	112±5	136±8	50±3	0.2±0.0	146±3	0.8±0.1	0.0±0.0	0.0±0.0	377±21**	85±9 *	19±1
	Drought	75±9	34±6	2.3±0.2	18±2	55±7	36±2	92±8	109±9	49±3	0.2±0.0	140±10	0.7±0.1	0.0±0.0	0.0±0.0	214±25	56±6	17±0
SIAL-169	Control	93±11	46±5	2.1±0.1	28±1**	53±5	45±4	98±9	127±9*	50±3	0.3±0.0	132±14	0.7±0.0	0.0±0.0*	0.0±0.0	599±39**	132±5 **	21±0 **
	Drought	73±7	34±3	2.2±0.1	21±2	43±4	37±3	80±6	101±7	51±1	0.3±0.0	129±5	0.7±0.0	0.0±0.0	0.0±0.0	219±16	75±3	18±0
SIC-17	Control	102±2**	45±1**	2.3±0.1	23±1**	46±2**	49±2**	96±3**	119±4**	48±1	0.2±0.0	129±3	0.9±0.0	0.0±0.0**	0.0±0.0**	530±47**	97±3 **	19±1 *
	Drought	67±4	27±1	2.5±0.1	16±1	34±1	32±2	65±3	81±3	47±1	0.2±0.0	127±3	0.8±0.0	0.0±0.0	0.0±0.0	168±15	55±4	17±0
SIC-2	Control	91±4**	38±1**	2.5±0.2	22±2**	42±2*	43±2**	85±4**	107±6**	47±1	0.3±0.0	142±2**	0.9±0.0	0.0±0.0	0.0±0.0	410±36**	93±5 **	20±1 **
	Drought	59±4	27±0	2.2±0.1	14±1	33±2	29±3	62±5	76±6	49±3	0.2±0.0	122±3	0.8±0.0	0.0±0.0	0.0±0.0	200±27	50±5	17±0
SPA-5	Control	81±4	46±3	1.8±0.1	21±2	52±9	40±4	91±11	112±#	48±2	0.2±0.0	141±5	0.8±0.1	0.0±0.0	0.0±0.0	427±11**	83±4 **	20±1 *
	Drought	69±8	36±5	2.0±0.2	18±2	43±2	34±4	77±5	96±6	50±2	0.2±0.0	132±3	0.7±0.1	0.0±0.0	0.0±0.0	216±12	58±5	18±1
TSA-792	Control	95±3**	54±4*	1.8±0.1	25±3	47±5	45±2*	92±7*	116±9*	48±2	0.3±0.0	130±5	0.8±0.1	0.0±0.0	0.0±0.0*	482±28**	104±5 **	20±0 **
	Drought	68±6	38±5	1.9±0.2	19±1	36±2	33±3	69±5	87±5	48±2	0.3±0.0	120±4	0.8±0.0	0.0±0.0	0.0±0.0	295±26	61±5	17±0
TSH-1188	Control	86±5*	53±4	1.7±0.1	30±2*	48±5	42±2	90±7	120±8	48±1	0.3±0.0	120±5	0.7±0.0	0.0±0.0	0.0±0.0	585±61**	122±11 **	20±1 **
	Drought	72±2	41±4	1.8±0.1	24±1	43±5	36±1	79±6	103±7	49±1	0.3±0.0	111±4	0.7±0.0	0.0±0.0	0.0±0.0	326±40	76±3	18±0

Statistical significance (Student's t-test) for the differences between control and drought treatments is indicated as follows: P<0.05*; P<0.01**. The means represent 6 replications ±/S.E.

Abbreviations: TLAP ×10^−2^, total leaf area per plant (m^2^ plant^−1^); LNP, leaves number per plant; ILA ×10^−2^, individual leaf area (m^2^); RDB, root dry biomass (g); SDB stem dry biomass (g); LDB, leaf dry biomass (g); SB, shoot biomass (g); TDB, total dry biomass (g); SLB, specific leaf biomass (g m^−2^); R/S, root/shoot ratio; PH, plant height (cm); LAR, leaf area ratio (dm^2^ plant^−1^); NAR, net assimilation rate (g dm^−2^ day^−1^); RGR, relative growth rate (g g^−1^ day^−1^); ARS, area of root system (cm^2^); RV, root volume (cm^3^); SD, stem diameter (mm).

Soil water deficit significantly (P<0.05) reduced leaf area per plant (TLAP), individual leaf area (ILA) and leaf number per plant (LNP) for most of the genotypes evaluated (Table 3). Significant reductions (P<0.05) were observed mainly for the LNP and PH variables in drought sensitive genotypes (Table 3).

**Figure 2:**
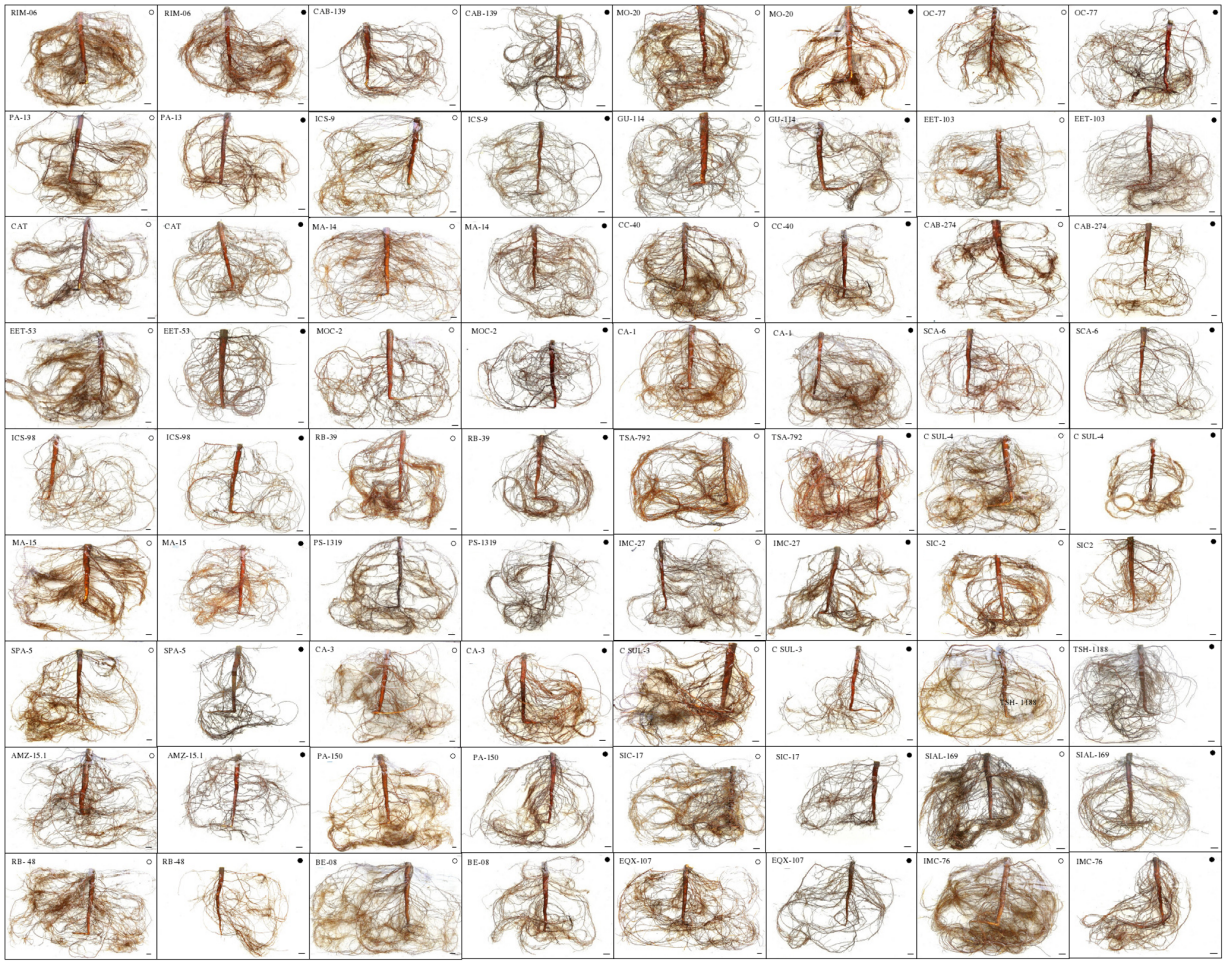
Photographs of roots for measurement of ARS of 36 genotypes of *Theobroma cacao* L. subjected to soil water deficit for 60 days. Control (○) water suppression (•). Scale: −2 cm.

In general, the cacao genotypes evaluated showed significant reductions (P<0.05) in stem diameter (SD), root volume (RV) and root area (ARS), with the exception of some tolerant genotypes (Table 3, Fig. 2). Overall in all genotypes tested, soil water deficit significantly reduced (P<0.05) growth variables such as SD, RV and ARS in 55, 75 and 81%, respectively, compared to the controls. Furthermore, no significant (P<0.05) intergenotypic reductions for R/S, SLB and LAR (Table 3) under water deficit conditions were observed. On the other hand, 42% of the evaluated genotypes showed significant reductions (P<0.05) for NAR and RGR, especially in sensitive genotypes, with decreases of 54 and 57%, respectively (Table 3).

### Macro and micro minerals nutrients

Soil water deficit significantly (P<0.01) reduced leaf macro and micro nutrient content for most of the evaluated genotypes, except for some tolerant and moderately tolerant ones (Table 4). Reductions in leaf content of N, P, K, Ca and Mg were found for 28, 22, 22, 69 and 56%, respectively, of all the genotypes subjected to soil water deficit.

**Table 4:** Macro and micronutrients leaf content evaluated in 36 cacao genotypes.

Genotype	Treatment	mg plant^−1^								
		N	P	K	Ca	Mg	Fe	Zn	Cu	Mn
AMZ 15.1	Control	884±23**	71±7	394±23	871±32**	397±11**	8±1	2.6±0.2*	0.6±0.0**	6.7±0.9*
	Drought	685±2	60±4	387±39	496±15	249±4	5±2	1.7±0.2	0.4±0.0	2.6±0.5
BE- 08	Control	790±46	50±1	249±9	856±22**	301±3**	7±0**	4.0±0.2*	0.6±0.0**	6.6±0.1**
	Drought	656±68	43±2	242±8	476±42	217±5	3±0	2.8±0.1	0.2±0.0	3.7±0.6
CSUL- 3	Control	759±91	59±4	465±8	686±40	376±7**	10±2	1.9±0.1*	0.5±0.0	3.2±0.1**
	Drought	800±31	54±2	436±27	567±24	296±7	7±3	1.4±0.1	0.6±0.0	2.4±0.0
CSUL -4	Control	1054±87**	78±9*	418±9	811±39**	411±23*	9±2	4.1±0.1**	0.6±0.1**	8.0±0.9
	Drought	636±12	51±8	356±34	526±19	240±30	5±1	3.1±0.0	0.1±0.0	5.1±1.0
CA-1	Control	618±42	51±7	256±30	842±45**	333±3**	7±1*	4.7±0.2**	0.6±0.1**	5.3±0.1
	Drought	720±82	63±6	243±15	446±38	221±13	2±1	2.4±0.2	0.1±0.0	3.5±0.9
CA-3	Control	711±81	47±3	299±28	999±31**	340±7**	10±1**	5.7±0.6*	0.8±0.1**	8.0±0.5**
	Drought	650±7	46±1	274±9	457±16	216±4	2±0	3.2±0.0	0.2±0.0	3.0±0.2
CAB-139	Control	1003±51**	84±6	457±19*	943±26**	468±6**	9±1**	3.1±0.8*	0.7±0.1*	5.0±0.7*
	Drought	590±84	65±5	334±33	582±46	309±3	4±1	1.8±0.0	0.2±0.1	2.2±0.0
CAB-274	Control	880±52	68±9	311±35	765±43	376±21*	5±1	3.0±0.0	0.5±0.0*	4.0±0.1**
	Drought	768±54	61±7	288±21	644±29	281±22	3±1	2.7±0.3	0.4±0.0	2.1±0.0
CAT	Control	719±77	58±2**	418±11**	600±23**	279±8**	5±0*	2.4±0.1	0.6±0.0*	3.8±0.5*
	Drought	561±3	44±1	279±21	447±14	220±5	1±0	2.2±0.2	0.3±0.0	2.6±0.1
CC-40	Control	880±37**	54±5	432±14**	840±72**	348±25*	8±1**	4.0±0.5*	0.9±0.1**	5.4±0.8*
	Drought	638±21	53±1	269±14	458±18	234±9	4±0	2.1±0.1	0.1±0.0	2.8±0.1
EET-103	Control	1032±73**	68±4	367±7	735±7**	353±11**	10±1*	3.0±0.1	0.6±0.0*	5.9±0.0**
	Drought	522±12	54±9	326±50	423±5	239±7	5±1	2.7±0.2	0.4±0.1	3.0±0.1
EET-53	Control	713±47	69±1**	353±13*	633±38	260±2	8±2*	2.7±0.1*	0.3±0.0**	3.9±0.5
	Drought	566±56	48±4	259±22	423±78	200±34	2±0	2.0±0.3	0.3±0.0	3.1±0.5
EQX-107	Control	967±105	75±7	393±30	839±117	400±49	6±2	1.4±0.1	0.4±0.0*	4.4±0.9
	Drought	860±27	62±2	374±10	617±37	295±21	5±0	1.8±0.2	0.6±0.0	3.2±0.0
GU-114	Control	928±108	75±6	332±24	787±60	338±43**	11±1	3.0±0.4	0.7±0.1*	5.5±0.3*
	Drought	744±83	64±4	275±15	636±41	271±6	5±1	3.0±0.2	0.4±0.0	4.0±0.3
ICS-9	Control	648±31	38±4	307±13	467±29**	242±10**	8±1*	2.4±0.1*	0.5±0.0*	2.5±0.3
	Drought	607±8	47±4	286±11	267±24	168±6	3±1	1.8±0.1	0.3±0.0	1.7±0.1
ICS-98	Control	691±88	42±6	304±43	539±22	343±23	15±1**	3.1±0.1**	0.3±0.0	4.2±0.2**
	Drought	629±137	48±4	259±25	507±115	279±48	4±0	2.3±0.0	0.3±0.0	2.5±0.1
IMC-27	Control	984±38*	73±3	376±11	773±46*	358±16	6±1	3.1±0.2	0.6±0.0**	5.7±0.3**
	Drought	711±59	64±5	357±28	526±43	299±31	6±2	3.4±0.0	0.4±0.0	3.8±0.1
IMC-76	Control	980±80**	87±9*	297±22	913±179*	384±55	7±2	4.2±0.5*	0.4±0.1	6.3±1.5*
	Drought	664±25	54±3	313±28	491±36	263±8	7±0	2.6±0.1	0.4±0.0	3.8±0.5
MA-14	Control	848±57	69±8	431±28	818±121	365±58	8±2	3.4±0.7	0.4±0.0	6.9±1.6
	Drought	820±39	60±5	357±24	765±45	310±11	9±1	3.4±0.1	0.5±0.0	6.5±0.7
MA-15	Control	742±50	62±3	299±21	795±27*	327±3	7±0*	3.1±0.0	0.5±0.0*	5.5±0.0**
	Drought	674±39	62±9	280±28	638±28	291±19	4±1	3.3±0.2	0.4±0.0	4.1±0.2
MO-20	Control	915±133	67±3	395±23	733±45*	343±14**	7±0	2.3±0.1*	0.6±0.0**	5.8±0.6
	Drought	805±49	63±5	330±24	548±23	266±4	6±2	3.4±0.7	0.4±0.0	6.2±1.3
MOC-2	Control	923±44	36±6*	322±6*	1092±63**	395±17**	11±0	1.6±0.1**	0.6±0.1**	5.0±0.2*
	Drought	839±26	67±2	370±10	558±39	258±15	10±3	1.1±0.0	0.2±0.0	3.1±0.6
OC-77	Control	375±45	30±2	220±10*	347±52	148±17	5±1	2.0±0.3	0.3±0.0**	1.7±0.3
	Drought	422±86	26±4	144±17	256±51	127±25	2±1	1.3±0.1	0.1±0.0	1.0±0.1
PA-13	Control	1006±114	71±8	357±26	790±35	308±18	9±1	3.2±0.3	0.5±0.0	6.1±0.3
	Drought	739±36	57±7	292±20	688±48	305±21	6±2	3.6±0.4	0.5±0.0	7.0±1.5
PA-150	Control	649±7	54±1	343±16	680±32	351±6*	11±1*	3.7±0.3	0.5±0.0*	6.3±0.6*
	Drought	704±89	59±7	244±33	546±63	277±16	4±1	2.5±0.4	0.3±0.1	3.8±0.3
PS-1319	Control	803±83	59±3*	399±13**	599±7**	284±4	10±1	2.5±0.0	0.5±0.1	6.3±1.5*
	Drought	585±28	49±1	293±17	518±3	254±11	7±2	2.3±0.1	0.3±0.0	3.5±0.3
RB-39	Control	835±37	55±10	352±27	958±59*	370±23	7±1	3.1±0.1	0.9±0.1*	4.9±0.4
	Drought	826±90	73±8	281±29	714±41	376±3	6±2	3.3±0.2	0.3±0.0	3.9±0.0
RB-48	Control	850±52	70±5	378±15	965±5**	440±11	15±0**	4.2±0.9*	0.7±0.1*	6.7±1.0*
	Drought	737±47	66±9	369±56	633±56	315±36	7±2	2.3±0.2	0.3±0.1	3.3±0.2
RIM-6	Control	826±47	67±1*	429±52	751±11**	340±27	7±2	4.1±0.0	0.7±0.0**	5.7±0.5**
	Drought	745±54	51±4	365±37	537±10	382±22	4±1	3.3±0.5	0.1±0.0	3.1±0.1
SCA-6	Control	941±13**	56±7	365±24	900±26**	384±7**	10±1**	3.2±0.2	0.6±0.0**	5.3±0.1**
	Drought	640±35	50±2	260±14	523±28	275±19	3±1	2.5±0.1	0.2±0.0	3.3±0.1
SIAL-169	Control	755±38	53±4	273±17	638±14	340±32	6±1	3.3±0.7	0.4±0.0	5.2±1.1
	Drought	606±79	53±2	266±30	589±63	297±20	5±1	2.6±0.0	0.4±0.0	4.3±0.4
SIC-17	Control	997±46**	61±4*	389±7**	907±68**	333±13**	7±1	4.0±0.1**	0.9±0.2*	5.8±0.3*
	Drought	667±30	43±3	190±17	447±60	234±12	4±0	2.5±0.2	0.4±0.0	3.9±1.0
SIC-2	Control	725±59	48±9	323±21	888±68**	326±26**	9±2*	3.9±0.1**	0.6±0.0**	4.9±0.1**
	Drought	568±48	49±2	279±17	414±10	196±4	2±0	2.3±0.1	0.1±0.0	2.4±0.2
SPA-5	Control	753±31	43±2	350±29	687±59*	267±20	11±2*	3.2±0.2**	0.5±0.0**	5.5±0.2**
	Drought	667±89	49±3	305±34	413±49	201±19	3±0	1.5±0.0	0.1±0.0	2.5±0.2
TSA-792	Control	772±14**	65±2	329±8	664±15*	312±11	5±0*	2.5±0.1	0.5±0.0*	4.8±0.5*
	Drought	571±38	54±8	315±29	424±61	257±49	2±1	3.0±0.7	0.3±0.0	2.9±0.2
TSH-1188	Control	653±28	45±3	254±2	752±23**	334±15**	10±2*	4.1±0.4*	0.8±0.0**	5.4±0.1**
	Drought	674±28	55±5	278±32	479±14	250±16	3±1	2.3±0.1	0.2±0.0	3.1±0.4

Statistical significance (Student's t-test) for the differences between control and drought treatments is indicated as follows: *P*<0.05*; *P*<0.01**. The means represent 6 replications ±/S.E.

Water deficit sensitive genotypes when subjected to soil water deficit showed the highest significant (P<0.01) reductions in leaf N, P and K content, compared to control plants (Table 4).

The vast majority of the genotypes evaluated also showed changes in foliar micronutrient content when subjected to soil water stress, except for tolerant genotypes (MA-14, PA-13 and SIAL-169). There were significant reductions (P<0.05) in foliar contents of Fe, Zn, Cu and Mn in 53, 50, 81 and 69% of the genotypes evaluated, respectively (Table 4).

### Enzyme activity

**Figure 3:**
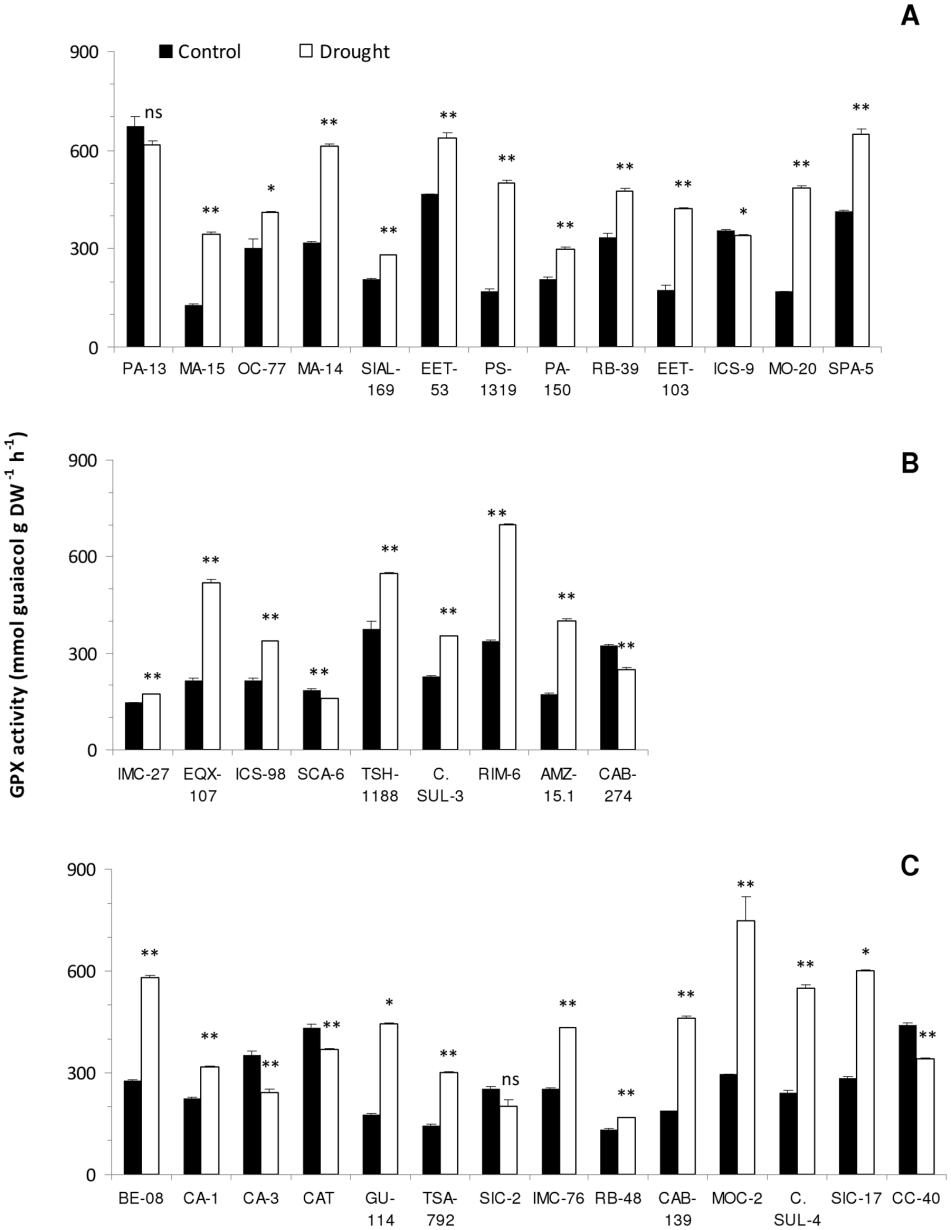
Activity of Guaiacol peroxidase (GPX) of *T.cacao* plants subjected to two watering regimes (well-watered and drought stress). A- Tolerant; B- Moderately tolerant; C- Sensitive genotypes. Open bars represent drought stress and closed bars represent well-watered. (⊤) - mean standard error. Number of replicates (n = 8), statistical significance for the differences between well-watered and drought stress treatments is indicated as follows: * P<0.05; ** P<0.01.

Overall, soil water deficit (drought) increased the activity of oxidative stress enzymes for most cacao genotypes evaluated, except for the tolerant genotype PA-13. The increase in peroxidase (GPX) activity was observed in 81% of the genotypes subjected to soil water deficit. Higher variations (P<0.01) were observed for tolerant genotypes (PS-1319, MO-20 and MA-15), which corresponded to increases in activity of 193, 188 and 170%, respectively, compared to controls. However, significant reductions (P<0.01) in these enzyme activities were observed for sensitive genotypes (CA-3, CAT and CC-40) and moderately tolerant genotypes (CAB-274, and SCA-6), under soil water stress which corresponded to reductions of 31, 15, 23, 23 and 13%, respectively, compared to controls (Fig. 3).

**Figure 4:**
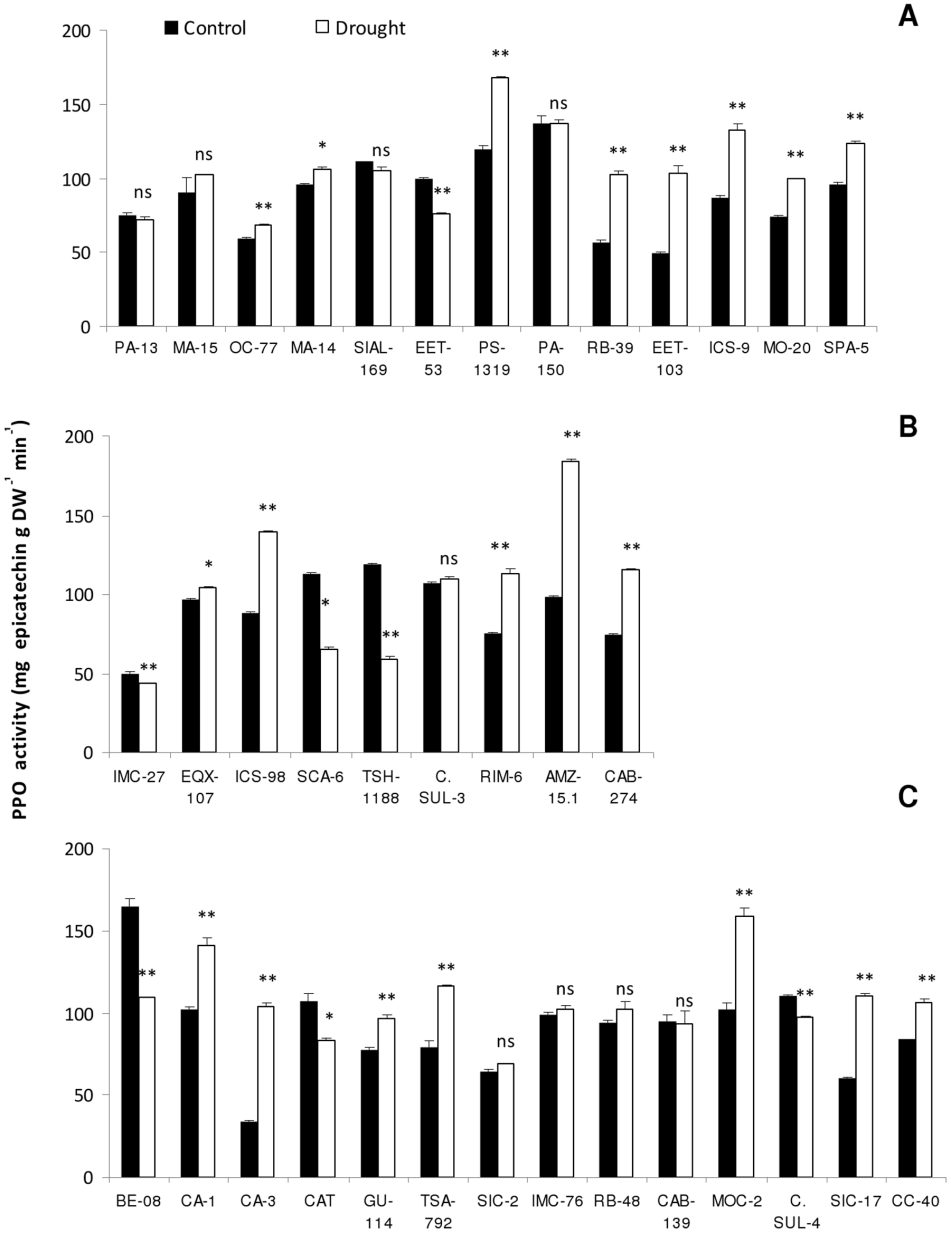
Activity of polyphenol oxidase (PPO) of *T. cacao* plants submitted to two watering regimes (well-watered and drought stress). A- Tolerant; B- Moderately tolerant; C- Sensitive genotypes. Open bars represent drought stress and closed bars represent well-watered plants. (⊤) - mean standard error. Number of replicates (n = 8), statistical significance for the differences between well-watered and drought stress treatments is Indicated as follows: * P<0.05; ** P<0.01.

Regarding polyphenol oxidase (PPO) activity, there were significant changes (P<0.01) observed in 75% of the studied genotypes under water stress. The highest values for the activity of PPO was found in moderately tolerant and susceptible genotypes (Fig. 4).

### Identification of tolerant genotypes based on multivariate analysis

**Figure 5:**
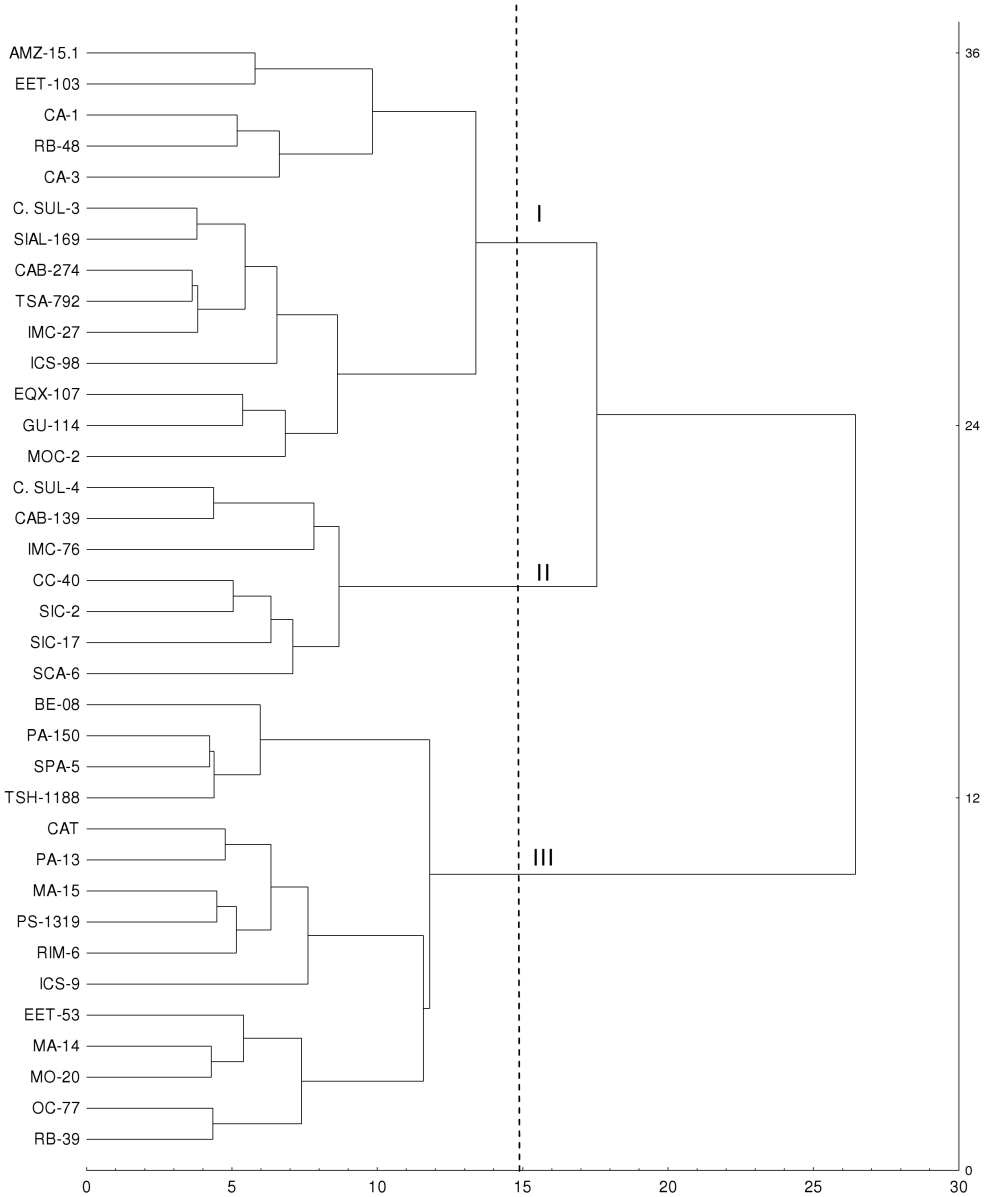
Cluster analysis of 36 genotypes of *Theobroma cacao* L. submitted to soil water deficit for 60 days based on the Euclidean distance from the difference between control and drought for growth variables, oxidative stress (GPX and PPO) and chemical composition evaluated using the hierarchical clustering method Ward (1963).

A multivariate analysis was performed to determine if the growth parameters, chemical composition and activities of oxidative stress (GPX and PPO) enzymes could provide information regarding selection of the most tolerant genotypes to water stress. Initially a cluster analyses based on the similarity of these variables was performed, using the differences (Δ) between control and water stressed plants within genotypes. The Δ values were used to construct a similarity matrix and a dendrogram was constructed based on similarity data (Fig. 5). The results showed the formation of three distinct groups (Fig. 5). The first group (I) was represented by 14 genotypes, the second (II) by seven and the third (III) by 15 (Fig. 5). There was a relationship between the groups formed and the number of significant variables for the different genotypes (Table 5). Furthermore, there was an association observed between the similarity, based on the analyzed variables and drought tolerance. Thus, genotypes PA-13, MA-15, OC-77, MO-20, PS-1319 and MA-14 were grouped as being tolerant to water stress, with lower Δ compared to their respective controls. They were part of the third group, whereas the second group was formed by CC-40, C. SUL-4, SIC-4 and SIC-17, considered non-tolerant to water deficit, had higher Δ in relation to their controls (Fig. 5).

**Table 5:** Number of significant variables and distinct groups of 36 cacao genotypes subjected to water deficit in the soil for 60 days based in the 28 variables evaluated.

Genotype	Total	Groups	Genotype	Total	Groups
PA-13	4	Tolerant	C.SUL-3	14	Moderately tolerant
OC-77	5	Tolerant	RIM-6	14	Moderately tolerant
MA-15	5	Tolerant	AMZ-15.1	15	Moderately tolerant
MA-14	6	Tolerant	CAB-274	15	Moderately tolerant
SIAL-169	7	Tolerant	CAT	16	Sensitive
PS-1319	8	Tolerant	GU-114	16	Sensitive
EET-53	8	Tolerant	BE-08	16	Sensitive
PA-150	9	Tolerant	CA-3	16	Sensitive
RB-39	9	Tolerant	TSA-792	16	Sensitive
ICS-9	10	Tolerant	CA-1	16	Sensitive
MO-20	10	Tolerant	SIC-2	17	Sensitive
SPA-5	10	Tolerant	IMC-76	18	Sensitive
EET-103	10	Tolerant	RB-48	19	Sensitive
IMC-27	11	Moderately tolerant	CAB-139	20	Sensitive
EQX-107	12	Moderately tolerant	MOC-2	21	Sensitive
ICS-98	12	Moderately tolerant	SIC-17	22	Sensitive
SCA-6	13	Moderately tolerant	C.SUL-4	22	Sensitive
TSH-1188	13	Moderately tolerant	CC-40	23	Sensitive

**Figure 6:**
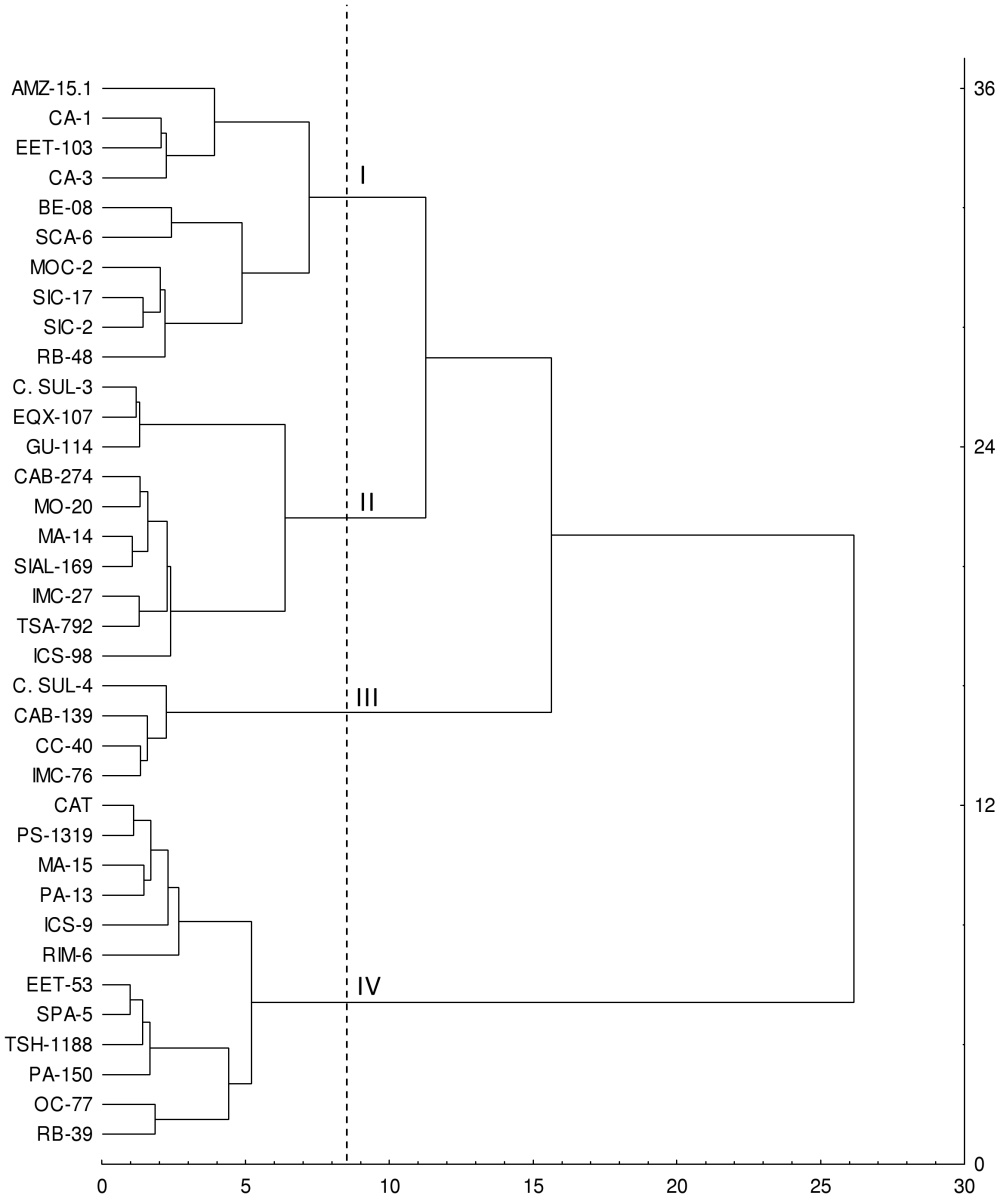
Cluster analysis of 36 genotypes of *Theobroma cacao* L. submitted to soil water deficit for 60 days, based on the Euclidean distance from the difference between control and drought for the variables TLAP, RDB, SDB, LDB, TDB, RGR, and leaf contents of Ca and Mg, using the method of hierarchical clustering Ward (1963).

Next, from the factor analysis and colinearity test, we observed that the variables TLAP, RDB, SDB, LDB, TDB, RGR, Ca and Mg had the greatest contribution on the formation of the first factor and showed no colinearity among them. By submitting the Δ data of the non collinear variables to a cluster analysis and performing a dendrograma, four main groups were formed (Fig. 6). These results were similar to those groupings observed when a cluster analysis was performed using all growth variables, oxidative stress (GPX and PPO) and chemical composition. Thus, it can be suggested that the eight non-collinear variables are sufficient to separate the contrasting *T. cacao* genotypes in relation to tolerance to soil water deficits tolerance.

**Figure 7:**
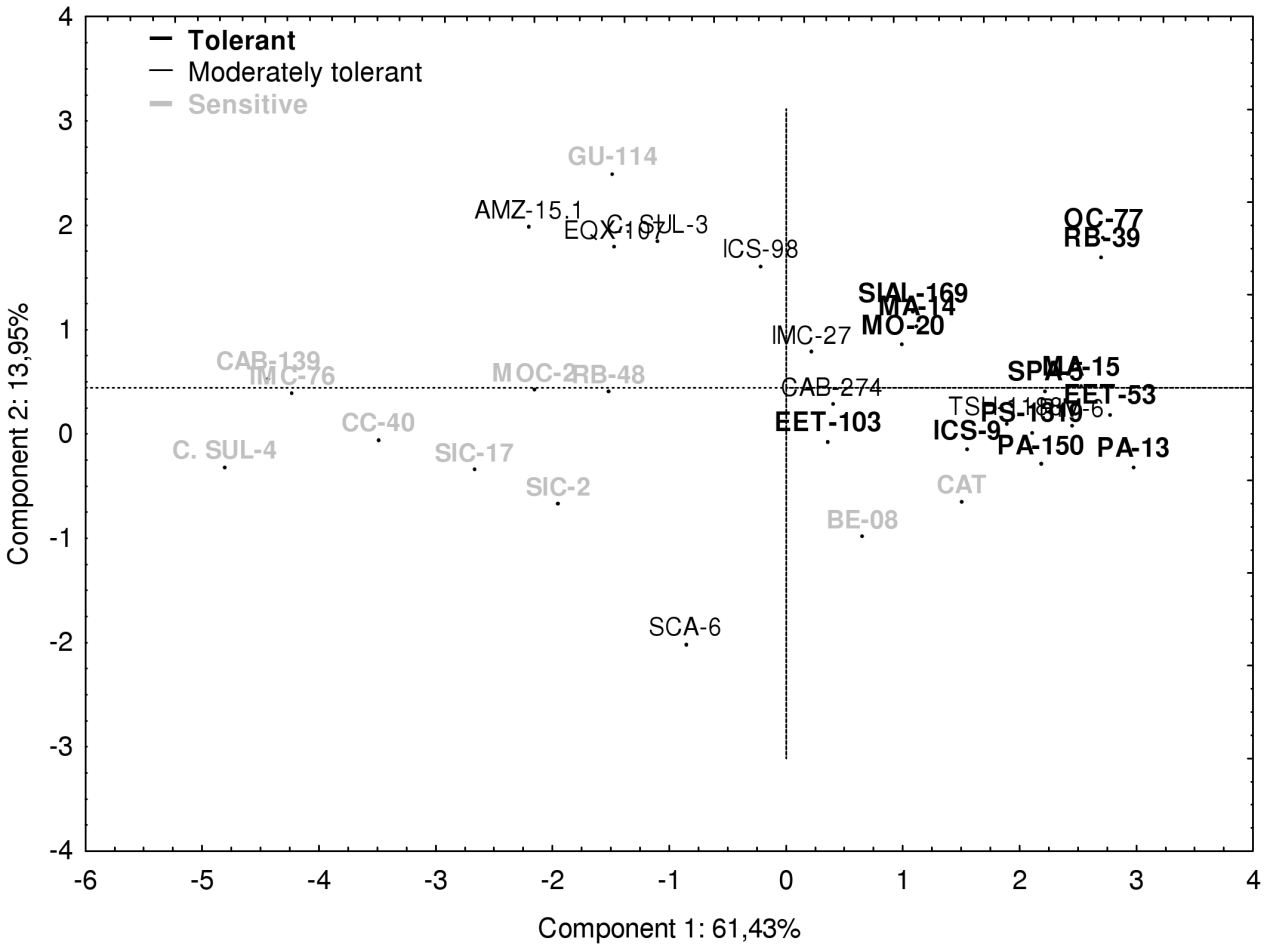
Principal components analysis of 36 genotypes of *Theobroma cacao* L. subjected to soil water deficit for 60 days, based on the difference between control and drought for the variables TLAP, RDB, SDB, LDB, TDB, RGR, and leaf contents of Ca and Mg.

Principal components analysis formed groups, separating the more contrasting *T. cacao* genotypes regarding tolerance to soil water deficit (Fig. 7). Furthermore, the results agreed with cluster analysis by the agglomerative method of Ward (49). The first and second principal component explained 61 and 14%, respectively, of the total variance with a cumulative eigenvalue of 75% (Table 6). From the eigenvectors values, we observed that the variables that had the higher contribution in the formation of the first component were, TDB, RGR, LDB and foliar Mg content while the variable SDB and TLAP had the highest contribution in the second component. The remaining components explained 11, 7, 3, 2 and 1%, respectively, of the total variance (Table 6).

**Table 6:** Eigenvalues and eigenvectors of the correlation matrix for the variables TLAP, RDB, SDB, LDB, TDB, RGR, and leaf contents of Ca and Mg in 36 cacao genotypes subjected to soil water deficit for 60 days.

Component	Eigenvalue	Cumulative %	Eigenvectors of correlation matrix							
			TLAP ×10^−2^	RDB	SDB	LDB	TDB	RGR	Ca	Mg
1	4.91	61.43	−0.33	−0.31	−0.3	−0.38	−0.43	−0.42	−0.25	−0.36
2	1.12	75.37	−0.46	0.24	0.61	−0.39	0.22	0.18	−0.33	−0.14
3	0.92	86.92	0.39	0.26	−0.18	0.26	0.11	0.04	−0.72	−0.38
4	0.56	93.93	−0.25	0.83	−0.42	−0.11	−0.03	−0.13	0.22	0.01
5	0.27	97.36	−0.01	0.03	−0.09	−0.14	−0.11	−0.14	−0.49	0.84
6	0.13	98.97	−0.67	−0.16	−0.16	0.67	0.18	0.02	−0.13	0.03
7	0.08	100	−0.1	−0.09	−0.34	−0.17	−0.29	0.87	−0.06	0.00

According to the first component, tolerant genotypes (Fig. 7) showed the greatest intergenotypic distinction. These genotypes had the lowest Δ values for linear combinations of the analyzed variables. Moreover, sensitive genotypes were grouped based on the high Δ values for variables with greater contribution in the formation of this component. These variables strongly contributed in the separation of tolerant and non-tolerant genotypes to soil water deficit.

### Gene expression

**Figure 8:**
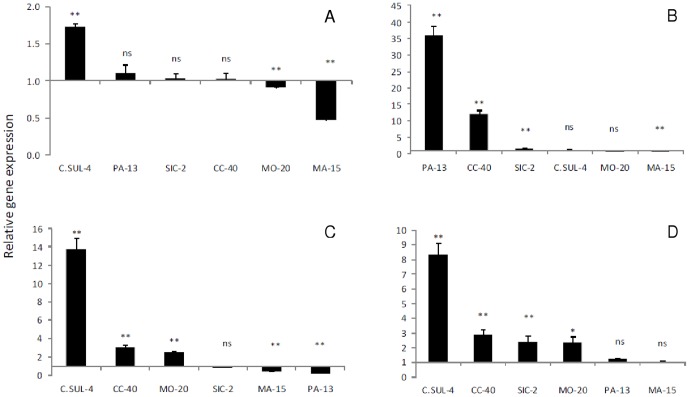
Expression of *psbO* (A) gene, *psbA* (B), *NCED5* (C), and *PP2C* (D) in plant leaves of 6 genotypes of *Theobroma cacao* L. subjected to soil water deficit for 60 days. 2^-ΔΔCt^ method. β-tubulin gene as a reference.

We observed increased expression of drought tolerance candidate genes in the studied genotypes. Genes associated with ABA biosynthesis and genes related to biosynthesis of proteins of PSII were expressed in genotypes considered as non-tolerant to soil water deficit and repression of these genes was observed for tolerant genotypes, compared to controls (Fig. 8). Furthermore, regarding the number of *psbO* transcripts, there was a significant two fold increase (P<0.01) in the expression of the non-tolerant genotype C. SUL-4, whereas for the tolerant genotypes MO-20 and MA-15 there was a significant suppression (P<0.01) by 0.9 and 0.5 times, respectively (Fig. 8 A). Furthermore, there was a significant increase (P<0.01) in the number of *psbA* transcripts for the tolerant genotype PA-13 and the non-tolerant genotypes CC-40 and SIC-2 of 36, 12 and 2 times, respectively, compared to controls, while MA-15 showed repression of that gene by 0.8 times (Fig. 8 B). A significant increase (P<0.01) in the expression of *NCED5* was found, mainly in non-tolerant genotypes C. SUL-4 and CC-40, which corresponded to 14 and 3 times, respectively, to that of control plants. Furthermore, for tolerant genotypes MA-15 and PA-13, we observed a significant suppression (P<0.01) by 0.4 and 0.2 fold, respectively, in the expression of that gene (Fig. 8C). Also, there was an over expression of *PP2C*, especially in non-tolerant genotypes C. SUL-4, CC-40 and SIC-2, with increases of 8, 3 and 2 times, respectively, while for tolerant genotypes PA-13 and MA-15 no significant increases were found (Fig. 8D).

## Discussion

Soil water shortage is considered a major limiting factor in the production of many crops throughout the world. Physiological, biochemical and molecular responses in plants subjected to drought can be used as selection criteria for crop tolerance to this abiotic stress [27], [20], [49]. In genotypes with no tolerance to drought, soil water deficit promotes significant alterations in growth and development, by affecting both shoots and roots dry biomass distribution. Studies with *Eucalyptus microtheca* grown under water stress conditions have shown reductions in root, stem, leaf and total biomass distribution, thereby affecting the root/shoot ratio [50]. Similar responses have been reported for *Hippophae rhamnoides* [51] and *Populus* spp. [52], [8], which also showed significant reductions in total biomass accumulation and root/shoot ratio.

Of the 36 *T. cacao* genotypes evaluated, sensitive genotypes showed the greatest damage at the leaf level when subjected to water deficit, with sharp reductions in TLAP, LNP and ILA. On the other hand, tolerant genotypes showed no alterations in these variables under water stress conditions (Table 3).

Reductions of TLAP, LNP and ILA promote, among other factors a decrease in photosynthesis and contributes significantly to the inhibition of plant growth [53]. In *T. cacao*, reductions in growth rates of leaf area and of total leaf area can be considered one of the earliest plant responses to stress as a result of the reduction in cell turgor and net photosynthetic rate [54], [55]. In clones of *Populus* subjected to cycles of soil dehydration and rehydration, changes in TLAP were explained by differences in the number of leaves and the further expansion of ILA [56].

Drought conditions induced significant reductions in RGR and NAR (42% for both variables) in the studied cacao genotypes (Table 3). It is known that, in tree species, in general, NAR and RGR are differently affected by low soil water availability, which indicates that responses to water stress are complex, heterogeneous and may be consistent with the geographical distribution of each species [57], [58].

In the present study, the cacao's responses to drought conditions in relation to height, SD, RV and ARS were quite varied among the genotypes, but the non-tolerant genotypes showed a marked reduction in the values of these variables. On the other hand, for drought tolerant genotypes these changes were not similar to results found in *Quercus* sp [59] and *Populus* sp [60], [61], [8]. The genotypes that showed marked reductions for the RV and ARS variables also showed decreased SB (Table 3, Fig. 2), suggesting that plants sensitive to water stress show reductions in both the root and the shoot growth. Furthermore, limitation of the root system of these genotypes influenced the absorption of water and nutrients, thereby affecting the plant water status. We have also observed that cacao genotypes tolerant to drought maintained a root growth similar to the control plants, showing higher amounts of fine roots (Fig. 8). In contrast, in genotypes that showed significant reductions in growth variables, the proportion of fine roots also showed reductions. Silva and Kummerow [62] found, under field conditions, that plants of *T. cacao* produced large numbers of fine roots (diameter <1 mm), which renewed quickly between one and 10 days, and growth were dependent on the frequency of rainfall. The dynamics of growth and renewal of roots, among other factors, can affect plant growth [63], [64]. Tschaplinski *et al.* [65] in studies with *Populus* found that the clones most tolerant to water stress showed phenotypic plasticity in relation to greater carbon allocation to the roots, favoring increased root density and, consequently, occupying a greater soil volume, thereby restoring the water balance in the plant.

The responses of plants to drought at the mineral nutrition level are still poorly studied [16], although mineral macro and micronutrients have specific functions and may be required in large amounts by plants [66]. In the present study water deficit resulted in significant decreases in the mineral nutrient contents of leaves, a similar response of mineral nutrient reduction was observed in *Fagus sylvatica* when subjected to drought [16]. The cacao genotypes that were more tolerant to soil water stress showed no significant differences in leaf N, P and K contents between water deficit and control (Table 4). Usually, high concentrations of N-NO_3_^–^ are deposited in the vacuole, contributing significantly to the maintenance of cellular turgor, thus conferring tolerance to drought conditions [66]. Furthermore, changes in P concentrations can have positive effects by increasing water use efficiency and stomatal conductance [67]. Moreover, under water stress, activation of several transcription factors and regulation of gene expression depend on phosphorylation of protein mediated by protein kinases [66]. For K, an essential macronutrient for plant growth and development, accounting for nearly 70% of nutrients in the cacao xylem sap [68], a decrease in foliar nutrient content was found mainly for sensitive genotypes that also showed significant reductions of TDB and NAR. Potassium acts to regulate osmotic potential, required for enzyme activity and protein and carbohydrate syntheses, and helps in the process of stomatal opening and closure, and participates in water relations and cell elongation. Potassium deficiency slows plant growth, promotes leaf chlorosis, necrotic spots and shortening of internodes [69], [66].

Although the content of macronutrients showed differences among genotypes, Ca and Mg content exhibited the greatest reductions with decreases of 69 and 56%, respectively (Table 4). However, tolerant genotypes maintained the content of these elements similar to controls. Maintaining high Ca and Mg content in these genotypes may have contributed to the increase in biomass and leaf area [66], activation of protein kinases, osmotic regulation and the opening and closing of stomata [20], [70]. On the other hand, the marked deficiency of Ca and Mg found in sensitive genotypes may have influenced the highly significant reduction in shoot biomass [71].

Under water stress conditions, plants may exhibit micronutrient deficiency [15] that causes damage at the metabolic cellular level, since micronutrients have an important role in the protection against oxidative stress and are involved in the regulation and activation of enzymes that remove ROS [18]. In this study, the effects of water stress reduced Fe, Zn, Cu and Mn content for most genotypes, indicating that water stress influenced the uptake of these micronutrients by the cacao plants. Furthermore, the deficiency of these minerals may have interfered in photosynthesis and nitrogen fixation [1], [72], and consequent biomass accumulation, and in the activities of peroxidases and polyphenol oxidases, enzymes responsible for elimination of ROS [18]. Micronutrients act as cofactors for enzymes of the antioxidative metabolism, Fe^2+^ for catalases and peroxidases [73], Zn for superoxide dismutase and other enzymes of the antioxidative metabolism [74], [75], [18], Cu for polyphenol oxidase, and Mn activates superoxide dismutase [18], enzymes contributing to drought tolerance in plants. It is suggested that in addition to water deficit *per se*, the reduction in area and volume of the root system contributes to the poor uptake and promotes the deficiency of these elements, aggravating the response of the genotypes to drought [75].

Under conditions of soil water deficit, plants tend to increase the production of ROS, as one of the first plant responses to stress, due to stomatal closure and reduction in CO_2_ fixation, which leads to excess excitation energy not being dissipated by the plant protection mechanisms [8], [76]. Most cacao genotypes in our study showed significant increases in GPX and PPO activities. It is inferred that Fe deficiency may have contributed to the reduction of GPX activity for some moderately tolerant genotypes (Fig. 3), since, as mentioned above, this element acts as cofactor of peroxidase enzymes [73]. Oxidative stress enzymes are activated to remove ROS, which can promote cell damage, senescence and leaf abscission under water stress conditions [76] and induce programmed cell death [25]. Polyphenol oxidase promotes removal of hydrogen peroxide (H_2_O_2_) [25], [13]. Studies have shown a relationship between changes in peroxidase activity and stress tolerance and this may be an adaptation mechanism of plant tissues to stresses [77], [78].

From the results of PPO activity it was not possible to separate cacao genotypes contrasting tolerance to soil water deficit. PPO enzymes are found in thylakoids and plastids, but there is not much information about the effects of changes in the activity of these enzymes during plant growth in response to water stress [79]. In most studies addressing the activity of PPO, there is a relationship of this enzyme to physiological damages. Polyphenol oxidase activity increases in response to different stresses [80], [81], [82].

Plants under abiotic stresses show changes in gene expression and regulation, in both the short and long term, as tolerance responses to unfavorable conditions [83]. In this study, cacao genotypes tolerant to water stress showed no changes in gene expression, contrasting with results found in *Arabidopsis thaliana* [84]. However, this is most likely due to the fact that the duration and intensity of the drought stress imposed in our study were applied gradually and over a longer period of time, which could be the reason transcription of some genes may have stabilized in tolerant genotypes (Fig. 8). The large accumulation of *psbO* transcripts for sensitive genotypes and repression for tolerant genotypes (Fig. 8A) suggests that its accumulation cannot be directly linked to drought tolerance, although the degradation of *psbO* protein probably destabilizes the oxygen evolution complex under drought conditions [37] and its reduction may limit plant growth and the concentration of other proteins encoded by both *psbA* and *PSBP* [36]. Moreover, the increase in the number of *psbA* transcripts, which encodes the D1 protein of the reaction center of PSII, may indicate that the protein is differentially expressed and easily damaged under water stress conditions [85].

Tan *et al.* [84] studying five genes of the NCED family in *Arabidopsis* reported that over a period of 35 h there was increased *NCED5* expression in flowers and leaves under water stress conditions. Chao *et al.* [86] found that Mg deficiency resulted in an increase in ABA concentrations in leaves of *Oryza sativa*. This was also found in the current study with the increased expression of *NCED5* in sensitive genotype C. SUL-4 (Fig. 8 C). However, there are few studies related to the function and expression of *NCED5*, mostly performed in *Arabidopsis*, with increased expression of this gene under stress conditions [84], [87]. Our results suggests that over expression of *PP2C* in genotypes susceptible to drought may indicate inactivation of protein kinases, and the consequent blocking of signal transduction in pathways dependent on ABA, phosphorylation, activation of transcription factors and expression of genes that confer drought tolerance [88], [35].

## Conclusions

Soil water deficit affected the majority of the physiological and biochemical variables as well as gene expression in the cacao genotypes evaluated in this study. Multivariate analysis showed that growth variables LDB, TDB, RGR and TLAP as well as the content of Mg in leaves were the most important variables in the separation of the genotypes as tolerant, moderately tolerant and sensitive to soil water deficit, therefore these traits are important in the selection of plants tolerant to drought.

## Supporting Information

S1 Table Difference (Δ) in values between control and drought plants for morphophysiological and biochemical variables, assessed on 36 genotypes of T. cacao.
(XLS)

S2 Table Factor analysis of 28 standardized variables, obtained from the difference (Δ) between the control plants (−0.1 to −0.5 MPa) and plants subjected to water stress (−2.0 to −2.5 MPa).
(XLS)

S3 Table Tolerance (TOL) and variance inflation factor (VIF) test for multicollinearity among variables included in the analysis.
(XLS)

S4 Table Activity of guaiacol peroxidase (GPX) and polyphenol oxidase (PPO) of T.cacao plants submitted to two watering regimes (well-watered and drought stress).
(XLS)

S5 Table Gene expression of psbO, psbA, NCED5, and PP2C in plant leaves of six genotypes of Theobroma cacao L. subjected to soil water deficit for 60 days. 2-ΔΔCt method. β-tubulin gene as a reference.
(XLS)
